# Effect of Cu- and Zn-Doped Bioactive Glasses on the In Vitro Bioactivity, Mechanical and Degradation Behavior of Biodegradable PDLLA Scaffolds

**DOI:** 10.3390/ma13132908

**Published:** 2020-06-29

**Authors:** Julian Bejarano, Aldo R. Boccaccini, Cristian Covarrubias, Humberto Palza

**Affiliations:** 1 Departamento de Ingeniería Química, Biotecnología y Materiales, Facultad de Ciencias Físicas y Matemáticas, Universidad de Chile, Beauchef 851, Santiago 8370456, Chile; 2 Department of Materials Science and Engineering, Institute of Biomaterials, University of Erlangen-Nuremberg, Cauerstrasse 6, 91058 Erlangen, Germany; aldo.boccaccini@ww.uni-erlangen.de; 3 Laboratory of Nanobiomaterials, Institute for Research in Dental Sciences, Faculty of Dentistry, University of Chile, Olivos 943 Independencia, Santiago 8380544, Chile; ccovarrubias@odontologia.uchile.cl; 4 Millennium Nuclei in Soft Smart Mechanical Metamaterials, Universidad de Chile, Santiago 8370456, Chile

**Keywords:** polymer scaffolds, bioactive glass particles, copper- and zinc-doped glasses, mechanical properties, degradation, in vitro bioactivity, bone repair

## Abstract

Biodegradable polymer scaffolds filled with bioactive glass particles doped with therapeutic metal ions are a novel and promising strategy to repair critical-sized bone defects. In this study, scaffolds based on a poly (D, L-lactide acid) (PDLLA) matrix filled with un-doped and Cu-, Zn- and CuZn-doped bioactive glass particles were produced by freeze-drying and a salt-leaching method. The effects of the doping and content of the glass particles (10 and 30 wt.%) on the morphology, compression properties, apatite formation, and degradation behavior of the scaffolds were evaluated. The scaffolds presented high porosity (~93%) with pores ranged from 100 to 400 μm interconnected by smaller pores and this porosity was kept after the glass particles incorporation. The glass particles reinforced the polymer scaffolds with improvements as high as 130% in elastic moduli, and further promoted the apatite formation on the scaffold surface, both properties depending on the amount and type of filler. The bioactive glass particles boosted the scaffold degradation with the PDLLA/un-doped glass scaffold showing the highest rate, but still retaining structural and dimensional integrity. Our findings show that the incorporation of un-doped and metal-doped bioactive glasses increases the mechanical strength, promotes the bioactivity and modifies the degradation profile of the resulting polymer/glass scaffolds, making them better candidates for bone repair.

## 1. Introduction

Bone defects with critical size derived from injuries, tumor resections, infections or genetic malformations do not heal spontaneously without a template, representing a significant challenge for physicians. The standard approach consisting in the harvest of bone from a donor site and afterward transplanting it into the defect site of the same patient is usually restricted due to the limited availability of autologous bone tissue and the significant morbidity caused at the extraction site [1]. Engineered templates, termed scaffold, have emerged as a strategy to face these challenges by providing mechanical support in the bone defect and promoting bone regeneration. Scaffolds should act as a temporary 3D material that permits and promotes cell processes (adhesion, proliferation and migration) and the subsequent formation of new tissue [2]. Additional considerations in the design and optimization of scaffolds intended for bone repair include appropriated biodegradability, permeability and mechanical support [3]. Among the different materials that can be considered, polymeric scaffolds are highlighted due to the easy processing and suitable biodegradation and biocompatibility [4,5]. However, bioactive components should further be added to stimulate the bone regeneration processes [6]. Bioactive particles are able to provide osteoconductivity (guidance for the growth of new bone) and stimulate both osteogenesis (bone tissue formation) and angiogenesis (blood vessel formation) [7]. In addition, it is greatly desired that these biomaterials have antimicrobial activity to reduce the risk of implant-centered infections caused by bacterial biofilms, which increase the patient morbidity and medical costs [8].

Composite materials based on biodegradable polymers filled with bioactive inorganic particles are an attractive strategy to develop multifunctional scaffolds for bone repair exhibiting the above-mentioned properties [9]. Bioactive glasses (BG) are well-recognized bioactive materials for bone repair applications due to their ability to form an apatite layer on their surface that promotes a direct bond to the living bone tissue [10,11,12]. Moreover, a number of inorganic ions can be incorporated into their glass structure—e.g., calcium (Ca), silicon (Si), phosphorous (P), zinc (Zn), copper (Cu), cobalt (Co), strontium (Sr), and magnesium (Mg) ions—which play important physiological roles in angiogenesis and bone metabolism as enzyme cofactors [13,14,15,16]. Since the discovery of the first silicate-based bioactive glass by Hench et al. [10], also called 45S5 Bioglass^®^ (45SiO_2_-25CaO-25Na_2_O-6P_2_O_5_, wt.%), silicate compositions have become important bioactive materials for bone regeneration due to their unique biological properties provided by their ionic dissolution products [14]. Si, Ca, Na and P ions are able to induce and stimulate the expression of genes related to cell differentiation and bone formation [14]. Additionally, Cu and Zn are essential trace elements for humans and can also be considered as relevant co-doping agents of silicate-based glasses for bone applications due to their important angiogenic, osteogenic and antibacterial activity [15,17]. A number of reviews and research studies have described the important biological role and benefits of cations like Cu^2+^ and Zn^2+^ for bone regeneration and bacterial control [14,17,18,19,20]. Our research group prepared bioactive glasses doped with Cu and Zn by the sol-gel method, which exhibited the formation of an apatite layer after immersion into a simulated body fluid (SBF) [21,22]. Additionally, these Cu- and Zn-doped glasses showed cytocompatibility and antibacterial activity [21,22].

However, in spite of the outstanding properties exhibited by these bioactive glasses, some difficulties such as their fragile behavior and poor ductility make difficult their implantation. These issues support the need to explore polymer/ceramic composites as the combination of the properties of biodegradable polymers and bioactive glasses will increase the performance for bone tissue regeneration [9]. In particular, these composite materials would not require a second surgery to remove them since during their degradation new bone tissue is formed and a gradual substitution of the implanted scaffold material would occur [9,23]. If these composite materials are properly designed, they should result in scaffolds with tailored physical and biological properties for specific bone applications [24]. For instance, dissolution of the bioactive glass phase would result in the formation of an apatite layer on the scaffold surface, thus providing the required osteoconductivity [24].

Resorbable polyester, such as polylactide acid (PLA), polyglycolic acid (PGA) and their copolymers, are highly considered as matrices for tissue engineering applications due to their approval from the US Food and Drug Administration (FDA) for clinical application [23]. Particularly, poly (D, L-lactide), termed PDLLA, is a biodegradable polymer extensively used for bone repair due to its remarkable combination of biocompatibility, mechanical strength and degradation profile [9,25]. In previous studies, Boccaccini et al. [26,27,28,29,30] developed composite scaffolds based on a PDLLA matrix filled with 45S5 Bioglass^®^ particles. These scaffolds showed increased cell attachment, proliferation and differentiation, as well as angiogenic potential [26,27,28,29,30]. Metal-doped bioactive glasses have been also incorporated into a PDLLA matrix. For instance, PDLLA scaffolds filled with Cu- and Zn-doped glass particles were recently prepared, showing cytocompatibility, antibacterial capacity and enhanced activity of the angiogenic and osteogenic biomarkers vascular endothelial growth factor (VEGF) and alkaline phosphatase (ALP), respectively [31].

In order to evaluate the suitability of these types of polymer scaffolds filled with metal-doped bioactive glasses as bone material candidates for clinical application, it is necessary to completely understand a set of key physicochemical properties, beyond the biological responses, that can provide input about the correct application of the implant material and its performance. Among these properties, it is important to evaluate the surface reactivity and its ability to attach to bone through the stimulation of biomineralization. Moreover, the study of the porous structure, mechanical properties and degradation profile would provide relevant input about the correct bone site that can be treated with the biodegradable material trying to match the properties of the natural bone.

Studies have described the effect of bioactive glass particles on the above-mentioned properties of poly (lactic acid)-based scaffolds [26,27,28]. Additionally, these studies have shown that the final properties of the composite scaffold will depend on the physicochemical characteristics of the glass particles and the percentage of incorporation into the polymer matrix [26,29,32,33]. In turn, despite the large amount of studies testing the effect of bioactive glasses doped with therapeutic ions on the biological response of different biodegradable polymers, the effect of the doping on the physicochemical behavior of the composites has been barely studied. Therefore, it becomes important to evaluate the effect of doping agents used in silicate glass particles on the final physicochemical properties of the composite scaffolds. In particular, the effect of metal-doped glasses, specifically Cu- and Zn-doped glasses, on important properties such as the porous structure, the capacity to form apatite, the mechanical properties and the degradation behavior of poly (lactic acid)-based scaffolds has not been studied to the best of our knowledge.

In this study, the effect of the incorporation of Cu-, Zn- and CuZn-doped bioactive glass particles (10 and 30 wt.%) on the ability to form apatite after immersion in SBF, the compression properties and degradation behavior of a PDLLA porous matrix were evaluated. The present study aims to understand the influence of these Cu- and Zn-doped bioactive glass particles and the co-doped glass with both Cu and Zn on the in vitro reactivity of PDLLA scaffolds, and provide insights for the design of these types of multifunctional scaffolds based on mechanical properties and degradation requirements.

## 2. Materials and Methods

### 2.1. Materials

A racemic poly(D, L-lactide) (PDLLA) polymer with a molecular weight of Mw = 406,000 g/mol, an inherent viscosity of 2.0 dL/g, and a density of 1.26 g/cm^3^ was provided by Corbion-Purac Biochem and used without further processing. Dimethyl carbonate (DMC; >99%; Sigma-Aldrich, Sheboygan Falls, WI, USA) was used as a solvent. Sodium chloride (NaCl) crystals with a particle size between 100 and 400 µm were used as a porogen for the polymer matrix. Bioactive glasses with compositions 60SiO_2_-25CaO-11Na_2_O-4P_2_O_5_ (mol.%) (BG), BG doped with 1 mol.% of CuO (CuBG) or ZnO (ZnBG), and co-doped with both metals (CuZnBG) were synthesized by the sol-gel method described in [22], and used as the bioactive phase in the preparation of the PDLLA composite scaffolds. Briefly, the sol was obtained from the hydrolysis of tetraethyl orthosilicate (TEOS; 98%; Aldrich, Wuxi, China), along with the addition of the other reagents sequentially every 45 min under constant stirring. Triethylphosphate (TEP; 99.8%; Sigma-Aldrich, Milwaukee, WI, USA), sodium nitrate (99.5%; Riedel-de Häen AG, Seelze, Germany) and calcium nitrate tetrahydrate (99%; Merck, Darmstadt, Germany) were used to obtain the composition of the un-doped glass. Additionally, copper nitrate trihydrate (99.5%; Merck, Darmstadt, Germany) and zinc nitrate tetrahydrate (98.5%; Merck, Kenilworth, NJ, USA) were further added to obtain the metal-doped glasses. An additional 60 min of mixing were let after the last reagent. The resulting sols were poured into high density polyethylene (HDPE) containers and stored for 3 days at room temperature (RT). Afterwards, the gels were dried at 60 °C for 3 days and 130 °C for 2 days. The dried gels were crushed using an analytic mill (Cole-Parmer, Vernon Hills, IL, USA) at 20.000 rpm for 30 s and subsequently heated at 700 °C for 3 h in an electric furnace with a heating rate of 5 °C/min and cooled to RT inside the furnace under oxidizing atmosphere (air). Finally, all the synthesized glasses were manually ground using an agate mortar and then sieved through a 400-mesh sieve (particle sizes < 37 μm). The resulting glass particles used in this study showed an irregular shape and average particle size of about 10 µm [22]. These glass chemical compositions were selected by their potential properties for bone regeneration as described in previous studies [22,31].

### 2.2. Production of PDLLA/Metal-Doped Bioactive Glass Porous Scaffolds

The neat PDLLA and PDLLA/Cu-, Zn- and CuZn-doped glass scaffolds were produced by both lyophilization and salt-leaching methods, as described elsewhere [31]. Briefly, a polymer solution was obtained by dissolving the PDLLA pellets in DMC solvent under magnetic stirring for 2 h at RT. A polymer weight to a solvent volume ratio of 8% (*w*/*v*) was used. The un-doped and metal-doped glass particles (10 or 30 wt.%) were added to the polymer solution, and this resulting mixture kept under mixing for 30 min. Then, it was sonicated for further 15 min to break up glass agglomerates and ensure a homogeneous distribution of the glass particles throughout the solution. These percentages of glass incorporation, 10 and 30 wt.%, were chosen after analyzing the results previously reported for composite materials based on PDLLA with similar concentrations of 45S5 glass and their potential to generate a relevant combination of these properties (apatite formation, degradation, and mechanical properties) [26,27,28]. After the glass incorporation, the porogen particles (NaCl crystals) was added to the suspension (NaCl to polymer weight ratio of 9:1) and stirred for 30 min. This resulting mixture was then poured into a polytetrafluoroethylene (PTFE) mold and maintained in a deep freezer at −80 °C for 3 h before freeze-drying for 24 h. After the lyophilization period to extract the DMC solvent, the scaffolds were immersed in distilled water for 48 h to completely dissolve the salt crystals and leave a porous structure. Water was changed each 4 h to avoid salt saturation. Finally, the resulting porous discs of 150 mm (diameter) × 4.5 mm (thickness) were dried at 30 °C for 48 h under vacuum. The scaffolds were cut using double-edged foil razor blades prior to material characterization. The composite materials based on PDLLA filled with 10 wt.% of glass particles were labeled as PLA/10-BG, PLA/10-CuBG, PLA/10-ZnBG and PLA/10-CuZnBG. Similarly, those with 30 wt.% of particles were called PLA/30-BG, PLA/30-CuBG, PLA/30-ZnBG and PLA/30-CuZnBG.

### 2.3. Characterization

#### 2.3.1. Scaffold Porosity

The theoretical porosities were calculated using the equation: *p =* 1 − (*ρ*/*ρ*_0_), where *ρ* is the apparent density and *ρ*_0_ is the density of the non-porous composite material [32].

The apparent density *ρ* was calculated from the mass to volume ratio of the scaffold specimens. The specimen volume was obtained from the sample dimensions (height, width, and depth) (*n* = 9). In addition, *ρ*_0_ was calculated from the rule of mixtures expressed in the equation: *ρ*_0_ = 1/((X_g_/*ρ*_g_) + (X*_p_*/ *ρ_p_*)) [32], where X_g_ and X*_p_* are the glass and polymer weight fractions in the composite, and *ρ*_g_ and *ρ_p_* are their densities, respectively.

The glass densities were measured using a pycnometer. Briefly, the weight of the dry and empty pycnometer was measured (W_1_), then 0.3 g of each glass sample were added to the pycnometer and weighed (W_2_). Water was added to the pycnometer containing the glass sample and weighed again (W_3_). Finally, the pycnometer was emptied, cleaned and filled only with water and its weight was recorded (W_4_). Measurements were repeated three times for each glass. The density was calculated from the formula: *ρ*_g_ = ((W_1_ − W_2_)/(W_4_ − W_1_) − (W_3_ − W_2_)) *ρ*_L_, where *ρ*_L_ is the density of water (1 g/cm^3^). The glass density results are presented as average value ± standard deviation. 

The polymer density (*ρ_p_* = 1.26 g/cm^3^) was obtained from the manufacturer. The pore morphologies and sizes were evaluated by scanning electron microscopy (SEM, FEI Quanta 250) at 20 kV. Before SEM analysis, scaffold specimenes were cut throught their cross section using a double-edged foil razor blade, attached on a carbon film and coated with a thin layer of gold using a SEM coating unit operated with a current of 30 mA for 60 s under an inert atmosphere.

#### 2.3.2. Mechanical Evaluation

Mechanical compression tests were performed at a loading rate of 1 mm/min using a testing machine Zwick Z050 with a load cell of 1 kN. Specimens with dimensions of (10 × 10 × 4.5) mm were tested and the stress–strain curves were obtained for each type of scaffold. The Young’s modulus was calculated from slope of the stress–strain curve before the onset of the yield point (elastic linear region), and the compression strength was presented as the stress measured at 10% relative deformation as described by the International Organization for Standardization (ISO) 844:2014 and American Society for Testing and Materials (ASTM) D1621:2000 standards [33,34]. The results were expressed as the mean ± standard deviation (SD) for *n* = 5.

#### 2.3.3. In Vitro Bioactivity Evaluation in Simulated Body Fluid (SBF)

In order to evaluate the in vitro apatite formation on the scaffolds, a SBF solution was prepared following the protocol described by Kokubo et al. [20]. An immersion step, whereby air was displaced within the foam specimens using vacuum for 10 min, was a critical preconditioning step for the in vitro studies. Otherwise, the foams floated and the test fluids could not interact effectively with whole scaffold’s surface. One specimen of each type of scaffold with dimensions (10 × 10 × 4.5) mm was immersed in 35 mL of SBF allowing a ratio of about 0.001 g/mL. Samples were kept in SBF for 1, 7, 14 and 28 days (one specimen per soaking time) under constant stirring at 120 rpm and 37 °C. For the specimens immersed for 14 and 28 days, their SBF solutions were changed every seven days to maintain a suitable ion concentration in the medium. After each soaking time, the specimens were removed from their containers and gently rinsed with distilled water. Then, the scaffold specimens were dried under vacuum at 30 °C for 24 h (Vacutherm, VT6060, Thermo Scientific, Wohlen, Switzerland). Finally, the dried scaffolds were characterized by attenuated total reflectance-Fourier transform infrared spectroscopy (ATR-FTIR), X-ray diffraction (XRD) and scanning electron microscopy with energy dispersive X-ray analysis (SEM/EDX) to evaluate the apatite formation on the scaffold surface. The XRD analysis was performed using a Bruker model D8 Advance x-ray diffractometer equipped with a lineal detector LynxEye, CuKα radiation λ = 1.5418 Å, nickel filter system and variable slit. The diffractometer was operated at 40 kV, 30 mA and a 2θ range of 20–50°, using coupled scanning with a step size of 0.02° s^−1^. The FTIR measurements were taken using an ATR-FTIR, Nicolet 6700 Thermo Scientific spectrometer (Waltham, MA, USA) in the spectral range 4000–400 cm^−1^ (data shown between 2000 and 400 cm^−1^ for better visualization of the bands of interest) with 64 scans at a resolution of 4 cm^−1^ and using a window of Caesium iodide (CsI). For the SEM analysis, the specimens were previously coated with a gold layer as described previously for porosity evaluation.

#### 2.3.4. Degradation Studies in Phosphate Buffer Solution (PBS)

A phosphate buffer solution (PBS) was used for the degradation studies to prevent the apatite formation avoiding misleading results, for instance, measurements of both weight and pH changes. A preconditioning step using vacuum was performed to ensure a complete immersion of the foams in PBS and its effective interaction with all the pore surfaces. Specimens of dimensions (10 × 10 × 4.5) mm were immersed in 10 mL of PBS and maintained for 1, 7, 14, 28, 60 and 120 days in a shaking incubator at 37 °C and 120 rpm. The PBS medium was changed every 30 days only for the samples soaked for 60 and 120 days. No PBS change was performed for the samples soaked for 1, 7, 14 and 28 days. After each immersion time, water absorption, weight loss and pH change were assessed. Briefly, these properties were calculated by weighing the samples before immersion in PBS (*W_i_*), immediately post-incubation performing previous blotting (Kimtech™ Science Kimwipes, Kimberly Clark wipes, Roswell, GA, USA) to eliminate the excess of PBS (*W_w_*) and after drying in a vacuum oven at 30 °C for 24 h (*W_d_*). The water absorption (WA, %) and weight loss (WL, %) of the specimens were calculated according to the equations: WA = ((*W_w_* − *W_i_*)/*W_i_*) × 100, and WL = ((*W_i_* − *W_d_*)/*W_i_*) × 100, respectively.

The result of WA and WL were expressed as mean ± standard deviation (SD) with *n* = 3.

## 3. Results and Discussion

### 3.1. Porosity and Pore Morphology

Porous PDLLA composite foams were prepared by lyophilization and subsequent porogen leaching. The glass densities used to calculate the density of the non-porous composite material had the following values for each type of bioactive glass composition: BG = 2.00 (±0.02), CuBG = 2.62 (±0.02), ZnBG = 2.59 (±0.01), and CuZnBG = 2.50 (±0.02) (g/cm^3^). The apparent density (*ρ*), the density of the non-porous composite material (*ρ*_0_) and the porosity of the scaffolds (*p*) values are shown in Table 1. The results listed in Table 1 show that both densities, *ρ* and *ρ*_0_, increased proportionally with the amount of glass particles incorporated into the PDLLA scaffold, which shows that incorporating a material with higher density, ceramic material like bioactive glass, into a polymer matrix (substituting polymer mass) would increase the density of the composite material proportional to the amount of the ceramic phase and its density as has also been reported previously for similar poly (lactic acid)/bioactive glass systems [28,35,36]. Scaffolds filled with the metal-doped glasses exhibited higher densities than the scaffold filled with the un-doped glass, which was also consistent with the higher density of the metal-doped glasses.

The calculated theoretical porosity showed high values for all the scaffolds prepared, reaching values around 93% with no significant effect of the incorporation of the glass particles. These porosities are suitable for bone regeneration applications allowing the cell and vascular growth within the full volume of the scaffolds, as reported previously [24,37]. 

**Figure 1:**
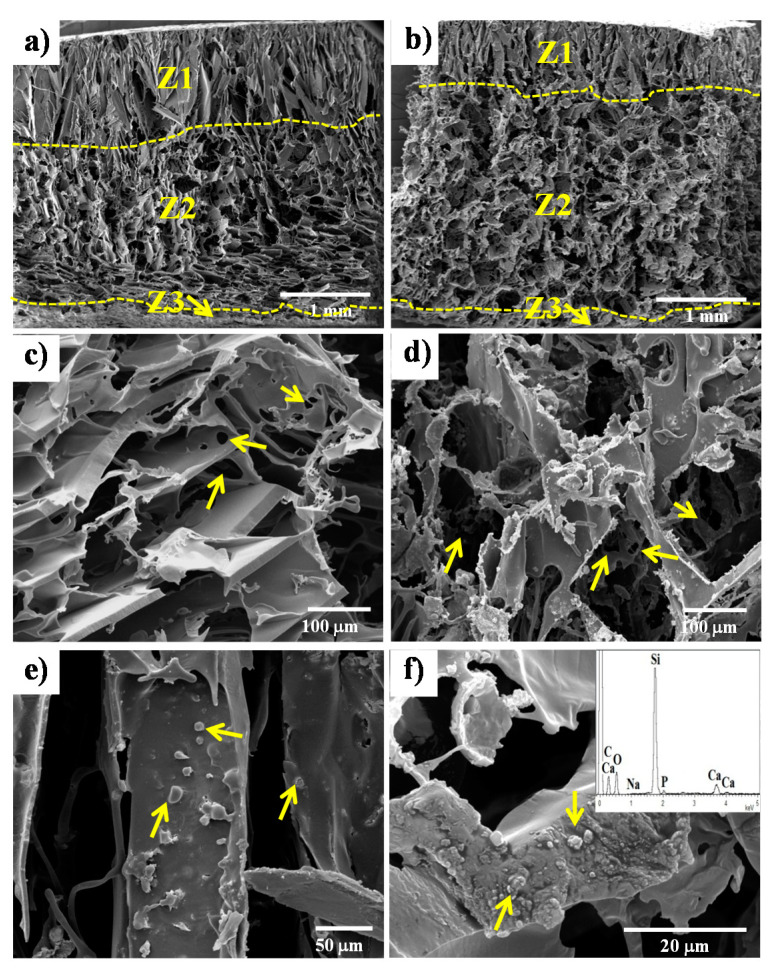
SEM images of the pore structure of the PDLLA scaffolds prepared by lyophilization and subsequent salt leaching methods. Porosity characterized by three zones (Z1, Z2 and Z3) through the cross-section of the scaffold samples: (**a**) PDLLA and (**b**) PLA/30-BG. Small pores (10–60 μm, indicated with arrows) interconnecting larger pores (100–400 μm) from Z2 are shown in (**c**) and (**d**) for PDLLA and PLA/30-BG, respectively. Glass particles exposed on pore wall surfaces (**e**) and embedded within the polymer matrix in the pore wall cross-section (**f**) are further shown for the PLA/30-BG composite scaffold by arrows. The insert of figure (**f**) shows the compositional analysis by EDX for the glass particles.

The pore morphology shown in Figure 1 presented some differences through the thickness of the samples represented by three zones called Z1, Z2, and Z3 (Figure 1a). Z1 is located at the top of the sample (around 25% of the thickness of the sample) and is characterized by a highly anisotropic structure composed of elongated pores, which is characteristic of the freeze-drying process (lyophilization), meaning that the NaCl particles sank from this zone before the sample will freeze. This type of tubular or elongated structures has also been obtained by other researchers using freeze-drying processes [28]. Z2 is the largest zone, occupying around 72% of the thickness, and is composed of more regular pores with sizes between 100 and 400 µm arising from the space left by the dissolved salt crystals. Moreover, these pores were interconnected with smaller pores with sizes between 10 and 60 µm (Figure 1c). The obtained pore sizes are in a range that has shown relevant effects on bone and vascular growth, facilitating the nutrients, oxygen and wastes transport as well as cell communication [9,38,39,40,41]. The last zone, Z3, it is a dense thin layer of around 20 µm produced by the wetting of the polymer solution on the mold surface. The same zones were observed for the composite scaffolds with 10 wt.% and 30 wt.% of glass particles, although the presence of the filler changed their dimensions as observed in Figure 1b for the PLA/30-BG sample. In this case, Z1 was reduced to 20% of the thickness while Z2 increased to 78%, which is likely due to the restriction of the salt crystals movement by the glass particles before freezing.

The pore sizes and interconnected structure in the composite scaffold (Figure 1d) did not show significant differences with those in the neat PDLLA scaffold (Figure 1c). However, the pore walls in both composite scaffolds with 10 wt.% and 30 wt.% (Figure 1d) glass particles had a more irregular or distorted morphology generated by the glass particles hindering the phase separation and crystallization during the cooling of the solvent, as has been reported by other studies in similar systems [27,42,43,44]. The SEM images in Figure 1e,f showed some particles both on the pore wall surface and embedded in the polymer matrix of the PLA/30-BG scaffold, respectively (indicated by arrows). A compositional analysis by EDX was performed to these particles (insert in Figure 1f) in order to confirm that those particles are certainly the glass particles. The presence of glass particles on the pore wall surface is important to confer both bioactivity to the PDDLA scaffold and an early release of therapeutic ions like Cu^2+^ and Zn^2+^ ions, while the bioactive glass particles embedded in the polymer matrix could permit long-term bioactivity as the scaffold degrades.

### 3.2. Mechanical Properties

**Figure 2:**
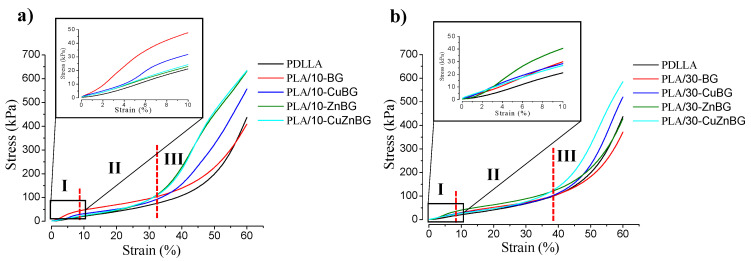
Stress vs. strain curve from the compression test for the scaffolds with (**a**) 10% and (**b**) 30% of the bioactive glass. Results from the neat PDLLA scaffolds are further shown. The inserts show the low strain region (elastic regime) and the dotted represents the limits for each zone of the curve (I, II and III).

The compression stress–strain curves of the scaffold samples containing 0, 10 and 30 wt.% glass particles are shown in Figure 2. All curves show the typical behavior of a compression test performed to a foam structure, as reported elsewhere [26,45]. At low stresses, an elastic linear zone

(I) Associated with the elastic deformation of the dense material forming the pore walls. This elastic deformation is reversible meaning that the polymer chains return to their equilibrium position when the stress is retired. After around 5–8% of strain, this elastic zone gradually disappears without a defined yield point because of the high porosity of these scaffolds. This transition indicates a second zone
(II) Characterized by a change in the stress variation with the deformation due to the bending of the pore walls and their plastic deformation [46]. Ending the plastic deformation, the pore structure collapses starting a third zone
(III) ICharacterized by the densification of the specimen at around 30–40% deformation. This denser specimen can support more load, and thus the compressive strength becomes notably higher with an exponential behavior.

By using these stress–strain curves it was possible to obtain two important mechanical properties of the scaffolds, such as the compressive strength and the Young’s modulus (elastic modulus). The inserts in Figure 2 further show the enlarged images of the first zone (I) or the elastic zone up to 10% of deformation from where these properties were calculated, as summarized in Table 2. The Young’s modulus was obtained by calculating the curve slope from the elastic zone and the compression strength was obtained from the maximum stress reached at 10% of deformation. The scaffold with 10 wt.% of the un-doped glass (BG) increased the compression strength of the PDLLA scaffold by 56% while scaffolds with a higher amount of this glass (30 wt.%) did not have a significant improvement on this property. In general, the incorporation of either Cu- and Zn-doped glass particles did not have any significant effect on these mechanical properties. The reinforcement effect of the particles on the polymer matrix could have been affected by particle agglomeration or changes in the glass particle–polymer interface due to the different properties of the un-doped and metal-doped particles, such as density and specific surface area [22]. The compression strength values obtained in this study for highly porous PDLLA scaffolds and their composites containing bioactive glass particles were in the same order of magnitude to those obtained previously in other studies for similar systems [26,29]. Regarding the elastic modulus, incorporation of 10 wt.% of glass particles into the PDLLA scaffold led to increases as high as 130%, compared with the neat PDLLA scaffold due to the higher modulus of the glass particles (ceramic material) and the load transfer mechanism, as have been reported in other studies [26,44,46]. A higher number of glass particles (30 wt.%) did not generate any additional improvement. The elastic moduli exhibited by the prepared composite scaffolds were smaller than the elastic modulus of the trabecular bone (around 10^5^ kPa) [47], because of the high porosity and the irregular interconnectivity of the pores in these scaffolds.

In general, better reinforcement or improved mechanical properties were not achieved by adding 30 wt.% of glass particles due to the greater likelihood of particle agglomeration and defects in the glass particle–polymer interfaces inside the irregular and distorted pore walls [29,43,48,49].

Considering the obtained mechanical properties, these type of scaffolds would be suitable for low-load-bearing applications, such as repair of small bone defects in orbital floor fractures or guided tissue regeneration membranes in periodontal disease [49,50,51].

### 3.3. Apatite Formation in SBF

**Figure 3:**
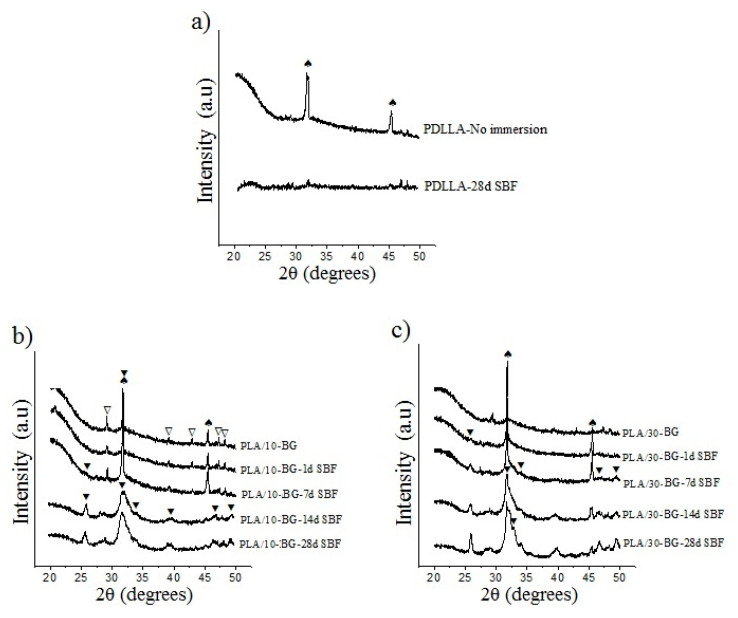
X-ray diffractograms showing apatite formation on the surface of the scaffolds with bioactive glasses (BG) after immersion in simulated body fluid (SBF). Scaffolds (**a**) neat PDLLA (**b**) PLA/10-BG and (**c**) PLA/30-BG. Crystal phases are labeled as: (⯆) apatite, (∇) CaCO_3_ (JCPDS 01-085-1108) and (♠) Halite (NaCl, JCPDS 5-0628).

The evaluation of the apatite formation on scaffold surfaces after 1, 7, 14 and 28 days of immersion in SBF was carried out using the XRD, ATR-FTIR and SEM techniques. Figure 3 shows the effect of the immersion in SBF on apatite formation evaluated by XRD for the neat PDLLA, PLA/10-BG and PLA/30-BG scaffolds.

The scaffolds did not show any characteristic peaks from the PDLLA matrix due to the amorphous structure of this polymer [28,52]. However, all scaffolds displayed the main peaks for Halita (NaCl, JCPDS 5-0628) at 2θ = 31.7° and 45.5°, associated with residues from the salt crystals used as a porogen. These peaks from NaCl crystals disappeared after immersion in SBF meaning the salt dissolution. Another phase present in the scaffolds was the calcium carbonate (CaCO_3_, JCPDS 01-085-1108) from the glass carbonation, although this phase was also dissolved during immersion. The neat PDLLA scaffold immersed for 28 days (Figure 3a) did not develop the characteristic peak of crystalline apatite at 2θ = 25.9°. However, a small peak around 2θ = 32° was observed indicating the formation of a calcium phosphate (CaP). The presence of CaP suggests the diffusion of ions from the SBF to the polymer surface forming this phase (biomineralization) [53].

The PDLA/10-BG sample showed the crystallization of apatite after seven days in SBF represented by the main peaks at 2θ = 25.9° and 32° (Figure 3b) likely arising from the glass particles exposed on the scaffold surface. For longer immersion times, the characteristic peaks of the apatite showed a larger intensity. Moreover, other peaks of the apatite phase at 2θ = 46.7° and 49.5° were observed. The crystalization of apatite increased with higher amount of glass particles into the polymer matrix, which was evidenced by the larger intensity of the apatite peaks in the XRD pattern of the PLA/30-BG scaffold compared to those of the PLA/10-BG scaffold (Figure 3c). Therefore, BG incorporation provides bioactivity to the polymer scaffold by formation of an apatite layer and this bioactivity proportionally increases with the amount of bioactive glass in the scaffold. Other studies have also found results consistent with this behavior [42,44].

**Figure 4:**
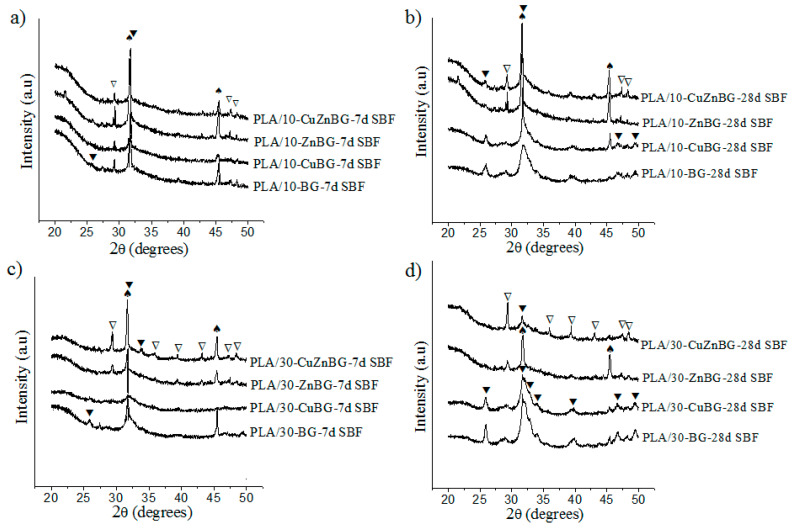
X-ray diffractograms showing apatite formation on the surface of the composite scaffolds with BG after immersion in SBF for 7 and 28 days. Scaffolds with 10% of glass particles after (**a**) 7 days and (**b**) 28 days of immersion in SBF. Scaffolds with 30% of glass particles after (**c**) 7 days and (**d**) 28 days of immersion in SBF. Crystal phases are labeled as: (⯆) apatite, (∇) CaCO_3_ (JCPDS 01-085-1108) and (♠) Halite (NaCl, JCPDS 5-0628).

Figure 4 shows the diffractograms for the scaffolds with 10 and 30 wt.% of the Cu-, Zn- and CuZn-doped glasses after immersion in SBF for 7 and 28 days. 

The metal-doped glasses also provided bioactivity to the PDLLA matrix depending on both the immersion time in SBF and amount of bioactive glass particles. Nevertheless, while the PLA/10-BG scaffold showed apatite formation after 7 days of immersion in SBF, the PLA/10-CuBG, PLA/10-ZnBG and PLA/10-CuZnBG scaffolds did not show clear evidence of the peaks of apatite at this immersion time (Figure 4a), thus showing a lower apatite formation by the incorporation of metal-doped glasses than that generated by the un-doped glass. The lesser degree of apatite crystallization on the scaffolds filled with Cu- and Zn-doped glasses compared to that of the scaffold with the un-doped glass can be explained by the greater chemical stability and density (higher network connectivity) of these metal-doped glasses compared to the un-doped glass. In a previous work, we showed a lower dissolution and ion release rate in the metal-doped glass particles and a subsequent delay or inhibition of the apatite formation as compared with the un-doped particles [22]. For instance, the un-doped glass released about 320 ppm of Ca after 7 days of immersion in SBF, while the Cu- and Zn-doped glasses released between 100 and 150 ppm of Ca having a direct negative effect on the apatite formation [22]. The decrease in the capacity to form apatite by the metal-doped glasses has been also explained by the competition for nucleation active sites between Cu^2+^ and Zn^2+^ with Ca^2+^ and PO^4−^ during the apatite formation process [22].

At 28 days in SBF (Figure 4b), all scaffolds with metal-doped glasses showed the peaks of the crystalline apatite including peaks at 2θ = 32.9°, 34.1°, and 39.8° for the PLA/10-BG and PLA/10-CuBG scaffolds. By increasing the amount of glass particles, these scaffolds showed increased bioactivity (Figure 4c,d). It was also observed that the presence of Zn-doped glasses in the scaffolds generated a lower apatite formation than the presence of Cu-doped glasses, which was also described for pure Zn- and Cu-doped glasses in our previous study [22]. The stronger apatite inhibition generated by Zn as a glass doping agent compared to Cu is a result of their different type of coordination (tetrahedral or octahedral) and bond geometries in the glass structure, which would affect the dissolution and ion leaching of the resulting glasses [22]. Zn is able to strongly bind to the glass network forming stable tetrahedral structures (Zn–O–Zn or Zn–O–Si) and more chemically stable glass structures [54,55,56]. Otherwise, no evidence of Cu–O–Si bond formation and no connection of Cu to the glass network has been found, meaning that Cu only participates as a glass modifier with octahedral coordination and weaker bonds in the glass network [57,58,59]. Therefore, the glass structure produced by the incorporation of Cu is less chemically stable, and hence with higher dissolution and ion release activity to form apatite than that generated by the incorporation of Zn [22]. For instance, in our previous work, the Cu and Zn release from this type of composite scaffolds was evaluated, finding that the release of Cu ions from the composite scaffolds was up to 30 times higher than the release of Zn ions. This demonstrates that the Cu-doped glasses are less chemically stable, and therefore with larger capacity for dissolution and ion release [31]. 

The NaCl and CaCO_3_ phases were also present, showing progressive dissolution with the immersion time.

**Figure 5:**
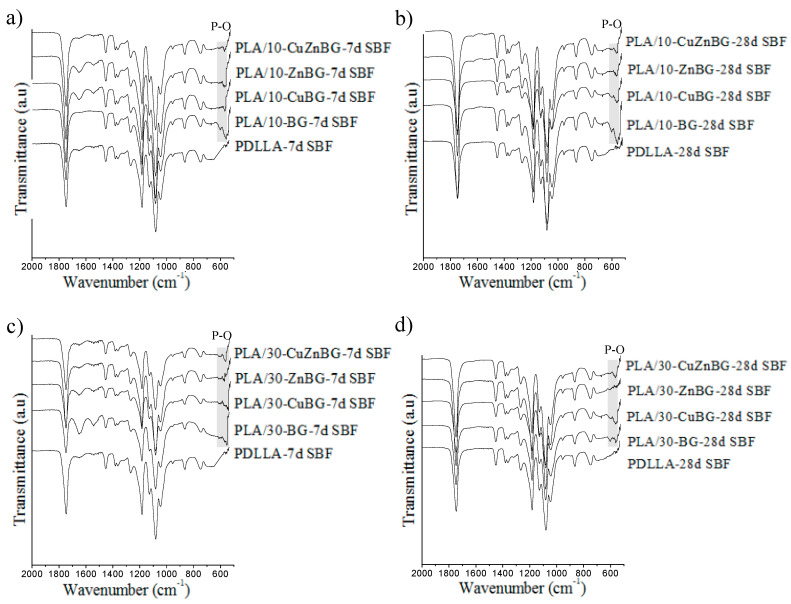
ATR-FTIR spectra indicating apatite formation on the surface of the composite scaffolds with BG after immersion in SBF for 7 and 28 days. Scaffolds with 10% of glass particles after (**a**) 7 days and (**b**) 28 days of immersion in SBF. Scaffolds with 30% of glass particles after (**c**) 7 days and (**d**) 28 days of immersion in SBF.

The apatite formation on the scaffolds evaluated by XRD was also confirmed using the ATR-FTIR technique after immersion in SBF for 7 and 28 days (Figure 5). All the ATR-FTIR spectra showed the characteristic bands of the PDLLA matrix at 750 and 865 cm^−1^ (CH bond), 1045, 1080, 1129, 1181 and 1267 cm^−1^ (—CO-O- ester group), 1383 and 1452 (CH_3_), and 1747 cm^−1^ (C = O carboxylic groups) [49,60]. For the glass-containing scaffolds, the band at 1045 cm^−1^ representing the Si–O–Si bond from the glass structure was overlapped with the ester group bands from PDLLA. PDLLA scaffolds did not show the characteristic bands of crystalline apatite at 560 and 602 cm^−1^ (P—O bond’s antisymmetric bending) after SBF immersion [61,62]. However, a small signal in this same region was observed, corresponding possibly to the biomineralization (CaP) described by the XRD results. Otherwise, all the composite scaffolds filled with the un-doped bioactive glass exhibited the characteristic bands of the crystalline apatite phase at 560 cm^−1^ and 602 cm^−1^ after 7 and 28 days of immersion in SBF being consitent with the XRD results. The delayed crystallization of apatite on the scaffolds with metal-doped glasses compared to those with the un-doped glass was also observed. Furthermore, the presence of the Zn-doped glass diminished even more the crystallization of apatite compared to the use of the un-doped, Cu- and CuZn-doped glasses, as was evident the disappearance of the band at 602 cm^−1^ for most of the spectra of scaffolds with the Zn-doped glass. As described above, the stronger apatite inhibition in Zn-doped glasses compared to Cu-doped glasses is originated by the difference of intermolecular forces and bond geometries of Cu and Zn in the glass network, which generates more stable glass structures with lesser dissolution and leaching activity for Zn-doped glasses [22]. Other studies have discussed that the strong inhibition of the apatite formation caused by Zn doping is due to the presence of Zn^2+^ ions in the active growth site of apatite which decelerate its crystallization [63,64].

**Figure 6:**
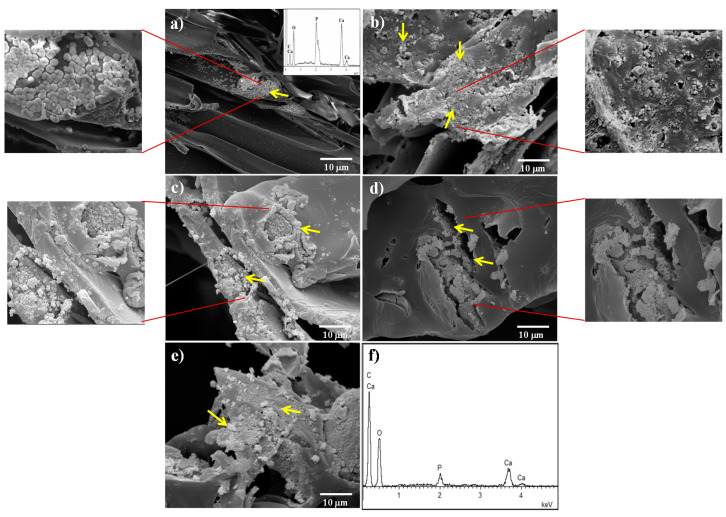
SEM images of the scaffold surface after immersion in SBF for 28 days: (**a**) PDLLA, (**b**) PLA/30-BG, (**c**) PLA/30-CuBG, (**d**) PLA/30-ZnBG, (**e**) PLA/30-CuZnBG and (**f**) EDX spectra of the apatite area. The yellow arrows show some apatite granules for the composite scaffolds and the calcium phosphate (CaP) precipitates on the neat PDLLA scaffold. SEM images with higher magnification show in more detail the apatite zones.

The effect of both the incorporation of bioactive glass particles into PDLLA scaffolds and the type of metal-doped glass on apatite formation upon immersion in SBF was evaluated using SEM. Figure 6 shows the surface of PDLLA, PLA/30-BG, PLA/30-CuBG, PLA/30-ZnBG and PLA/30-CuZnBG scaffolds after 28 days in SBF.

The PDLLA scaffold after 28 days in SBF (Figure 6a) presented mainly a smooth surface similar to that showed before immersion in SBF (Figure 1c). However, some scattered zones with small precipitates like apatite granules or globoids with needle-like crystals on the surface were observed. These biomineralized zones were composed of Ca and P according to the EDX (Figure 6a), being consistent with the XRD and FTIR results. These apatite granules have also been described by other studies on similar PLLA scaffolds after immersion in SBF for 28 days [53]. The PLA/30-BG scaffold (Figure 6b) showed a remarkable reactivity on the pore walls characterized by the rapid formation of apatite granules with needle-like crystals on the surface. The chemical composition of these apatite granules analyzed by EDX showed a spectrum without the presence of Si and with different intensities ratio between the Kα peaks of Ca and P compared with the spectrum of the composite scaffold before SBF (Figure 1f). The EDX analysis after SBF showed an estimated Ca/P ratio (atomic ratio%) of about 1.6 for the apatite zones. Polymer scaffolds filled with the Cu-, Zn- and CuZn-doped glasses showed surfaces with lesser degree of apatite-like granules formation, which can be due to a lower surface reactivity from the metal-doped glasses compared to that showed by the un-doped glass [22,31], confirming thus the results obtained by XRD and FTIR analysis. Nevertheless, all composite scaffolds showed reactivity in the areas around the glass particles. For instance, Figure 6c shows the surface of the PLA/30-CuBG scaffold with the formation of apatite granules around the glass particles. PLA/30-ZnBG (Figure 6d) scaffold also showed reactivity around glass particles, but not all the particles exhibited apatite formation due to the lower dissolution and leaching activity of this Zn-doped glass. Finally, PLA/30-CuZnBG (Figure 6e) also showed surface reactivity and apatite formation. The estimated Ca/P ratio (atomic% ratio) determined from the EDX analysis of the apatite zones on these scaffolds filled with metal-doped glasses was found about 2, indicating probably an apatite with a different stoichiometry due to differences in the dissolution rate, ion release profiles and ion adsorption, as described elsewhere [22]. Cu and Zn were not observed in the EDX spectra of the scaffolds filled with metal-doped glasses (data not shown), which can be explained by the low concentration of these elements as CuO and ZnO in the glass structure (1 mol.% or ~1.3 wt.%) and the low release of Cu and Zn from these types of scaffolds (less than 10 ppm for Cu and 1 ppm for Zn up to 21 days), according to our previously reported study [31]. This low concentration and release of Cu and Zn make difficult to detect these ions in the apatite layer, which is mainly based on Ca and P, and the concentrations of Cu and Zn are expected to be below the regular detection limit of EDX (about 0.1 wt.% or 1000 ppm) [65].

Based on the results in Figure 6, it is possible to conclude that the apatite formation on the composite scaffolds depended on the leaching activity and dissolution of the bioactive glass particles. In particular, glass particles exposed on the pore surface react first because of their direct contact with SBF. The glass dissolution over time can generate holes in the glass particle–polymer interfaces allowing the contact of the SBF with the embedded glass particles, which provides long-term bioactivity.

The results from XRD, ATR-FTIR and SEM/EDX analyses showed in general that bioactive glass inclusions promote apatite formation on PDLLA scaffolds after immersion in SBF. The leaching activity level, type and amount of apatite of the composite scaffolds depended on both the presence of Cu and Zn ions and the amount of bioactive glass particles incorporated. For instance, in scaffolds with 30 wt.% of bioactive glass the extent of apatite formation, classified from high to low, was found in scaffolds: PLA/30-BG, PLA/30-CuBG, PLA/30-CuZnBG, and PLA/30-ZnBG. For future studies, it would be interesting to study the incorporation of both the un-doped glass particles and some Cu-, Zn- or CuZn-doped particles into the same composite scaffold to allow the combination of increased initial apatite formation from the un-doped glass and delayed apatite formation and sustained therapeutic/antibacterial activity from the metal-doped glasses, which will help to advance on the design of tailored multifunctional scaffolds for bone regeneration.

### 3.4. Degradation Behavior

The degradation behavior of the scaffolds was evaluated after immersion in PBS for 1, 7, 14, 28, 60 and 120 days measuring the weight loss and pH changes. Before the immersion treatment, a preconditioning step using a vacuum was performed to ensure the effective interaction of the PBS with all pore surfaces. All the scaffold samples had a large water absorption, between 550–750%, compared to their initial weights, which is a result consistent with their high percentage of interconnected porosity (around 93%).

**Figure 7:**
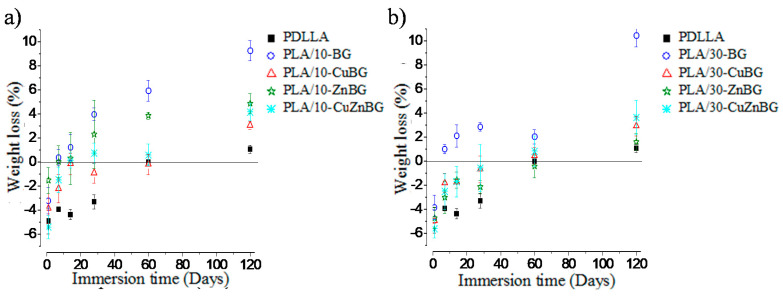
Weight loss of scaffolds samples after immersion in PBS for 1, 7, 14, 28, 60 and 120 days. Scaffolds with (**a**) 10 wt.% and (**b**) 30 wt.% glass particles.

Figure 7 shows the weight loss of the scaffolds based on the neat PDLLA and the composites with 10 wt.% and 30 wt.% of glass particles during the immersion in PBS. All the scaffolds presented initially a negative percentage of weight loss, which meant a higher final weight than the initial weight of the dry sample due to the trapped water that was strongly absorbed inside very small pores or capillaries of the polymer and the glass particles [29], or that was interacting with the polymer chains. This trapped water was difficult to extract during the drying process at 30 °C for 24 h after the immersion period. Moreover, even drying the samples for three days, no significant difference in the extraction of trapped water was observed. However, the weight loss for all scaffold samples increased over time indicating that the scaffolds were losing weight because of degradation. For instance, PDLLA scaffold started with a negative weight loss of ~4.9% and after 60 days of immersion the weight loss became 0%, and after 120 days this was of 1.1%. These values indicated distortion of the porous structure during the first 60 days allowing the extraction of the trapped water, and a slight weight loss (1.1%) by degradation at 120 days. Other studies have described a weight loss for PDLLA scaffolds between 1% and 2% during immersion of around 70 days associated with the generation of oligomers and a subsequent surface erosion [28,29].

**Figure 8:**
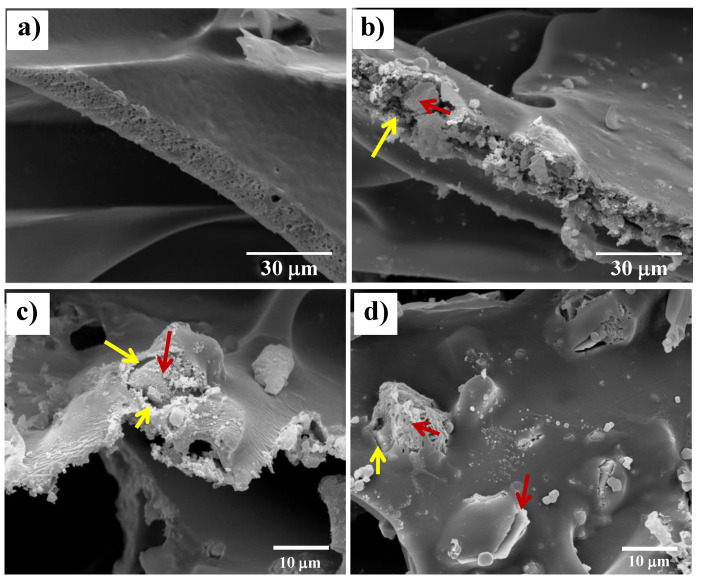
SEM images evaluating the differences in the degradation of the pore wall of (**a**) PDLLA, (**b**) and (**c**) PLA/10-BG, and (**d**) PLA/10-CuBG scaffolds, after 120 days of immersion in PBS. Glass particle–polymer interfaces are indicated with the yellow arrow and a glass particle with the red arrow.

The PLA/10-BG composite scaffold presented a negative weight loss after the first day of immersion, but at 7 days it exhibited a positive value of weight loss, reaching 9.3% at 120 days. This meant that this scaffold lost mass mainly by the partial dissolution of the BG particles, along with the contribution of the polymer degradation (around 1.1% of weight loss at 120 days), as described above. Additionally, it has been discussed in other studies that the presence of bioactive ceramic particles could also promote the polymer degradation by hydrolysis due to the higher absorption and retention of water along with the distortion of the pore wall in the glass particle–polymer interface, thus increasing the contribution of the polymer degradation to the total degradation of the composite scaffold [66,67,68]. Figure 8a,b show the difference in the pore walls between the neat PDLLA and PLA/10-BG scaffolds after 120 days of immersion in PBS, confirming the distortion of the pore wall and the formation of glass particle–polymer interfaces in the PLA/10-BG scaffold. Koort et al. [68] also found that the incorporation of glass particles into PDLLA generated higher degradation as distorted interfaces surrounding the glass particles were observed, which favor the polymer hydrolysis due to the greater surface available to react with the medium. 

Scaffolds incorporating 10 wt.% of Cu-, Zn- and CuZn-doped glasses also lost more weight than the neat PDLLA scaffold. However, the weight loss exhibited by the scaffolds filled with the metal-doped glasses was lesser than that of the scaffolds containing the un-doped glass particles because of the lower leaching activity and dissolution of the metal-doped glasses, as discussed above regarding previous studies [22,31,63,64]. For instance, PLA/10-CuBG scaffolds showed a surface with lesser degree of degradation than that of the PLA/10-BG scaffold at 120 days of immersion in PBS as evaluated by SEM (Figure 8c,d). The degradation behavior of polymer scaffolds containing therapeutic metal-doped glasses has not been evaluated before; therefore, this study represents an important advance in the knowledge of these composite systems to gain a better scaffold design and to achieve a more accurate prediction of their reactivity and degradation behavior.

Scaffolds containing 30 wt.% glass particles (Figure 7b) had the same tendency of weight loss than scaffolds containing 10 wt.% particles. In particular, the PLA/30-BG scaffold showed the highest percentage of weight loss (10.4%) at 120 days of immersion in PBS. However, scaffolds with 30 wt.% metal-doped glass particles had a lower weight loss (~3.5%) at 120 days. A controlled low weight loss until 120 days would be beneficial to maintain the porous structure and mechanical properties throughout the period when cells occupy the inner pores of the scaffold, the cellular matrix is generated and mineralization occurs (4–6 weeks, approximately) [29,69,70].

**Figure 9:**
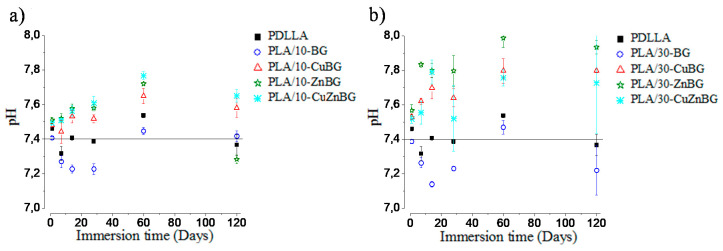
pH changes in phosphate buffer solution (PBS) after immersion of the scaffolds for 1, 7, 14, 28, 60 and 120 days. Scaffolds with (**a**) 10 wt.% and (**b**) 30 wt.% glass particles.

The degradation of scaffolds in PBS is associated with the release of byproducts which could change the pH of the local medium. For instance, PDLLA degrades in oligomers with carboxylic acid groups (lactic acid) [49], which diminish the physiological pH (pH = 7.4). On the other hand, bioactive glasses degrade releasing ions such as Ca^2+^ and Na^+^, which generate an increase of the pH [11]. pH changes have a significant effect on cell viability. Thus, it is important to evaluate the pH during the biomaterial degradation. Figure 9 shows pH changes in PBS (pH = 7.4) after the immersion of the scaffolds for 1, 7, 14, 28, 60 and 120 days. 

Figure 9a shows the pH change in PBS after the immersion of the neat PDLLA scaffold and the composite scaffolds with 10 wt.% glass particles. The neat PDLLA scaffold diminished the pH to 7.3 at 7 days of immersion, but at longer times the pH was around 7.4–7.5. The scaffold containing the un-doped bioactive glass (PLA/10%-BG) also had a slight decrease in pH up to 7.2 for 7, 14 and 28 days of immersion, which is due to the release of acid oligomers from the pore wall or glass particle–polymer interfaces [27]. At 60 and 120 days, the pH values were increased until 7.4–7.5, which was generated by exchanging of Ca^2+^ and Na^+^ ions from the glass surface with H^+^ from the medium as previously reported [22]. The ions coming from the glass particles play a role as a buffer to balance the acid byproducts from the polymer hydrolysis. Scaffolds containing 10 wt.% of the Cu-, Zn- and CuZn-doped glass particles (Figure 9a) increased the pH values up to 7.8 at 60 days, thus showing a higher alkaline effect with the incorporation of these metal-doped glasses. This higher alkaline effect is possibly due to the release of Cu^2+^ and Zn^2+^ ions to the PBS, forming copper and zinc hydroxides, such as Cu(OH)_2_ and Zn(OH)_2_, increasing thus the pH [71]. At 120 days, the pH showed a small decrease because of the acid products produced during the polymer degradation. The relatively small pH changes at early times and the pH balance generated by the degradation of the glass and polymer components coming from the neat PDLLA scaffold or composites containing 10 wt.% glass particles did not induce toxicity to stem cells at 10 days of incubation as was previously described in other study [31].

The relevance of the glass particles to modify the pH of the surrounding fluid is confirmed in Figure 9b, where more significative effects on the pH due to the greater degree of degradation and release of alkaline ions and acid products from scaffolds containing 30 wt.% of filler were observed. Although the pH reached acid and basic values about 7.1 and 8.0, respectively, during the period evaluated, it is worth noting that a balance was allowed between the alkaline ions released from the glass particles and the acid products from the polymer scaffold. This buffer effect could indicate that in dynamic conditions like those observed in the human body, it would be possible to have a physiological balance to avoid cytotoxicity by alkalosis or acidosis. In vivo or dynamic studies would be necessary for a more relevant assessment of these effects. For instance, previous in vitro cell culture tests [31] showed that a glass amount of 30 wt.% produced a cytotoxic effect at 10 days of incubation.

**Figure 10:**
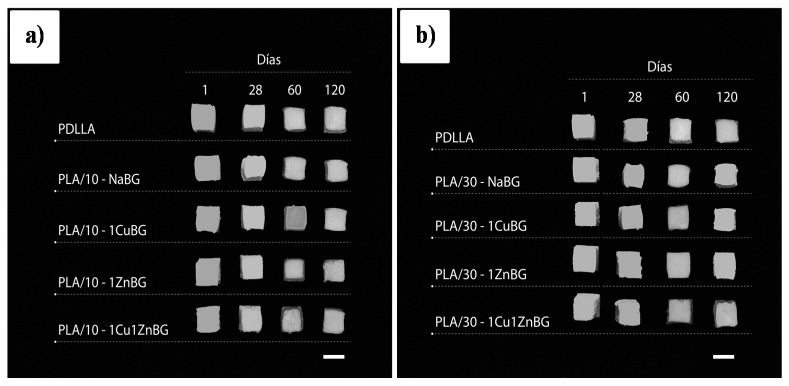
Photograph showing the appearance of the scaffold samples after immersion in PBS for 1, 28, 60 and 120 days. Bar scales: 10 mm.

The above-described weight loss and pH change results confirmed a relevant effect of the presence and type of bioactive glass. Additionally, significant changes in the dimensions and porous structure of the scaffold samples until 120 days were not observed, as shown in Figure 10, which was consistent with the low weight loss described above (Figure 7). This structural stability would be helpful to maintain the initial mechanical support for the growth of new bone tissue. 

## 4. Conclusions

Polymer scaffolds with 10 wt.% and 30 wt.% of un-doped and Cu-, Zn- and CuZn-doped bioactive glass particles were prepared and characterized to evaluate the effect of the doping with these therapeutic metal ions and the amount of glass particles on their in vitro bioactivity, compression and degradation behavior. The incorporation of these glass particles imparted in vitro bioactivity to PDLLA scaffolds forming apatite on their surface after immersion in SBF. Moreover, the incorporation of some glass particles at certain percentages improved the compression properties of the resulting composite scaffolds, as was evidenced, for instance, for the scaffold PLA/10-BG. The degradation behavior evaluated by the weight loss in PBS showed that these scaffolds can retain almost 90% of their initial weights after 120 days of immersion in PBS. It was also observed that the scaffold samples keep their dimensions and porous structure, which will be useful to support the growth of new bone tissue. Scaffolds did not significantly change the pH of the medium, which should avoid cytotoxic effects. All these properties depended on both the presence of the metal ions in the glass structure and the amount of glass particles incorporated into the porous polymer matrix. Despite the scaffold with 30 wt.% of the un-doped glass showed the highest in vitro bioactivity and degradation rate as well as improved mechanical strength which is useful for bone repair, the scaffolds with copper-doped glasses, i.e., either CuBG or CuZnBG, would be even better candidates to be clinically tested since they presented an important combination of apatite formation, mechanical strength and degradation profile. Additionally, the presence of therapeutic/antibacterial ions can provide additional activity to stimulate bone regeneration and avoid implant-centered infections. This study enabled the gaining of insights necessary to design novel therapeutic composite scaffolds for bone regeneration with improved bioactivity, mechanical properties and structural integrity.

## Figures and Tables

**Table 1:** Densities and porosity of the scaffolds.

Scaffold Sample	*ρ* (g/cm^3^)	*ρ*_0_ (g/cm^3^)	*p* (%)
Neat PDLLA	0.083	1.26	93.4
PLA/10-BG	0.086	1.31	93.5
PLA/30-BG	0.090	1.42	93.7
PLA/10-CuBG	0.091	1.33	93.2
PLA/30-CuBG	0.102	1.49	93.2
PLA/10-ZnBG	0.095	1.33	92.8
PLA/30-ZnBG	0.106	1.49	92.9
PLA/10-CuZnBG	0.099	1.33	92.5
PLA/30-CuZnBG	0.112	1.48	92.4

*ρ* apparent density; *ρ*_0_ = density of the non-porous composite material; *p* = theoretical porosity of the scaffold.

**Table 2:** Mechanical properties of the scaffolds.

Scaffold	Compressive Strength at 10% Deformation (kPa)	Young’s Modulus (kPa)
PDLLA	30 ± 3	2.3 ± 0.3
PLA/10-BG	48 ± 7	5.3 ± 0.3
PLA/30-BG	29 ± 4	3.1 ± 0.3
PLA/10-CuBG	32 ± 4	4 ± 1
PLA/30-CuBG	29 ± 3	3.5 ± 0.4
PLA/10-ZnBG	24 ± 3	2.8 ± 0.6
PLA/30-ZnBG	41 ± 6	5 ± 1
PLA/10-CuZnBG	26 ± 3	2.8 ± 0.6
PLA/30-CuZnBG	27 ± 3	2.8 ± 0.5

## References

[1] Stevens M.M. (2008). Biomaterials for bone tissue engineering. Mater. Today.

[2] Mohamad Y.D., Bretcanu O., Boccaccini A.R. (2008). Polymer-bioceramic composites for tissue engineering scaffolds. J. Mater. Sci..

[3] Wagoner J.A.J., Herschler B.A. (2011). A review of the mechanical behavior of CaP and CaP/polymer composites for applications in bone replacement and repair. Acta Biomater..

[4] Sabir M.I., Xu X., Li L. (2009). A review on biodegradable polymeric materials for bone tissue engineering applications. J. Mater. Sci..

[5] Kroeze R.J., Helder M.N., Govaert L.E., Smit T.H. (2009). Biodegradable Polymers in Bone Tissue Engineering. Materials.

[6] Boccaccini A.R., Erol M., Stark W.J., Mohn D., Hong Z., Mano J.F. (2010). Polymer/bioactive glass nanocomposites for biomedical applications: A review. Compos. Sci. Technol..

[7] Holzapfel B.M., Reichert J.C., Schantz J.-T., Gbureck U., Rackwitz L., Nöth U., Jakob F., Rudert M., Groll J., Hutmacher D.W. (2013). How smart do biomaterials need to be? A translational science and clinical point of view. Adv. Drug Deliv. Rev..

[8] Campoccia D., Montanaro L., Arciola C.R. (2013). A review of the clinical implications of anti-infective biomaterials and infection-resistant surfaces. Biomaterials.

[9] Rezwan K., Chen Q.Z., Blaker J.J., Boccaccini A.R. (2006). Biodegradable and bioactive porous polymer/inorganic composite scaffolds for bone tissue engineering. Biomaterials.

[10] Hench L.L., Splinter R.J., Allen W.C., Greenlee T.K. (1971). Bonding Mechanisms at the Interface of Ceramic Prosthetic Materials. J. Biomed. Mater. Res..

[11] Jones J.R. (2013). Review of bioactive glass: From Hench to hybrids. Acta Biomater..

[12] Arcos D., AVallet-Regí M. (2010). Sol-gel silica-based biomaterials and bone tissue regeneration. Acta Biomater..

[13] Mouriño V., Cattalini J.P., Boccaccini A.R. (2012). Metallic ions as therapeutic agents in tissue engineering scaffolds: An overview of their biological applications and strategies for new developments. J. R. Soc. Interface.

[14] Hoppe A., Güldal N.S., Boccaccini A.R. (2011). A review of the biological response to ionic dissolution products from bioactive glasses and glass-ceramics. Biomaterials.

[15] Hoppe A., Mouriño V., Boccaccini A.R. (2013). Therapeutic inorganic ions in bioactive glasses to enhance bone formation and beyond. Biomater. Sci..

[16] Habibovic P., Barralet J.E. (2011). Bioinorganics and biomaterials: Bone repair. Acta Biomater..

[17] Schuhladen K., Stich L., Schmidt J., Steinkasserer A., Boccaccini R., Zinser E. (2020). Cu, Zn doped borate bioactive glasses: Antibacterial efficacy and dose-dependent in vitro modulation of murine dendritic cells. Biomater. Sci..

[18] Cacciotti I. (2017). Bivalent cationic ions doped bioactive glasses: The influence of magnesium, zinc, strontium and copper on the physical and biological properties. J. Mater. Sci..

[19] Lakhkar N.J., Lee I.-H., Kim H.-W., Salih V., Wall I.B., Knowles J.C. (2013). Bone formation controlled by biologically relevant inorganic ions: Role and controlled delivery from phosphate-based glasses. Adv. Drug Deliv. Rev..

[20] O’Neill E., Awale G., Daneshmandi L., Umerah O., Lo K.W.-H. (2018). The roles of ions on bone regeneration. Drug Discov. Today.

[21] Palza H., Escobar B., Bejarano J., Bravo D., Diaz D.M., Perez J. (2013). Designing antimicrobial bioactive glass materials with embedded metal ions synthesized by the sol-gel method. Mater. Sci. Eng. C Mater. Biol. Appl..

[22] Bejarano J., Caviedes P., Palza H. (2015). Sol–gel synthesis and in vitro bioactivity of copper and zinc-doped silicate bioactive glasses and glass-ceramics. Biomed. Mater..

[23] Armentano I., Dottori M., Fortunati E., Mattioli S., Kenny J.M. (2010). Biodegradable polymer matrix nanocomposites for tissue engineering: A review. Polym. Degrad. Stab..

[24] Verrier S., Blaker J.J., Maquet V., Hench L.L., Boccaccini A.R. (2004). PDLLA/Bioglass s composites for soft-tissue and hard-tissue engineering: An in vitro cell biology assessment. Biomaterials.

[25] Li S.M., Garreau H., Vert M. (1990). Structure-property relationships in the case of the degradation of massive aliphatic poly- (-hydroxy acids) in aqueous media, Poly (DL-lactic acid). J. Mater. Sci. Mater. Med..

[26] Blaker J.J., Maquet V., Jérôme R., Boccaccini A.R., Nazhat S.N. (2005). Mechanical properties of highly porous PDLLA/Bioglass composite foams as scaffolds for bone tissue engineering. Acta Biomater..

[27] Maquet V., Boccaccini A.R., Pravata L., Notingher I., Jérôme R. (2003). Preparation, characterization, and in vitro degradation of bioresorbable and bioactive composites based on Bioglass-filled polylactide foams. J. Biomed. Mater. Res. A.

[28] Maquet V., Boccaccini A.R., Pravata L., Notingher I., Jerome R. (2004). Porous poly (a-hydroxyacid)/Bioglass composite scaffolds for bone tissue engineering. I: Preparation and in vitro characterisation. Biomaterials.

[29] Blaker J.J., Nazhat S.N., Maquet V., Boccaccini A.R. (2011). Long-term in vitro degradation of PDLLA/bioglass bone scaffolds in acellular simulated body fluid. Acta Biomater..

[30] Tsigkou O., Hench L.L., Boccaccini A.R., Polak J.M., Stevens M.M. (2007). Enhanced differentiation and mineralization of human fetal osteoblasts on PDLLA containing Bioglass(R) composite films in the absence of osteogenic supplements. J. Biomed. Mater. Res. Part A.

[31] Bejarano J., Detsch R., Boccaccini A.R., Palza H. (2017). PDLLA scaffolds with Cu- and Zn-doped bioactive glasses having multifunctional properties for bone regeneration. J. Biomed. Mater. Res. Part A.

[32] Sultan S., Mathew A.P. (2018). 3D printed scaffolds with gradient porosity based on a cellulose nanocrystal hydrogel. Nanoscale.

[33] ISO (2004). ISO 844-Rigid Cellular Plastics-Determination of Compression Properties.

[34] ASTM D (2010). 1621, Standard Test Method for Compressive Properties of Rigid Cellular Plastics.

[35] Ashby M., Shercliff H., Cebon D. (2007). Materials: Engineering, Science, Processing and Design.

[36] Boccaccini A.R., Maquet V. (2003). Bioresorbable and bioactive polymer/Bioglass^®^ composites with tailored pore structure for tissue engineering applications. Compos. Sci. Technol..

[37] Gerhardt L.-C., Widdows K.L., Erol M.M., Burch C.W., Sanz-Herrera J.A., Ochoa I., Stämpfli R., Roqan I.S., Gabe S., Ansari T. (2011). The pro-angiogenic properties of multi-functional bioactive glass composite scaffolds. Biomaterials.

[38] Houmard M., Qiang F., Saiz E., Tomsia A.P. (2013). Sol-gel method to fabricate CaP scaffolds by robocasting for tissue engineering. J. Mater. Sci. Mater. Med..

[39] Henno S., Lambotte J.C., Glez D., Guigand M., Lancien G., Cathelineau G. (2003). Characterisation and quantification of angiogenesis in β-tricalcium phosphate implants by immunohistochemistry and transmission electron microscopy. Biomaterials.

[40] Chen Q., Zhu C., Thouas G.A. (2012). Progress and challenges in biomaterials used for bone tissue engineering: Bioactive glasses and elastomeric composites. Prog. Biomater..

[41] Sundelacruz S., Kaplan D.L. (2009). Stem cell- and scaffold-based tissue engineering approaches to osteochondral regenerative medicine. Semin. Cell Dev. Biol..

[42] Blaker J.J., Gough J.E., Maquet V., Notingher I., Boccaccini A.R. (2003). In vitro evaluation of novel bioactive composites based on Bioglass-filled polylactide foams for bone tissue engineering scaffolds. J. Biomed. Mater. Res. A.

[43] Zhang R., Ma P.X. (1999). Poly(alpha-hydroxyl acids)/hydroxyapatite porous composites for bone-tissue engineering. I. Preparation and morphology. J. Biomed. Mater. Res..

[44] Conoscenti G., Carfì Pavia F., Ciraldo F.E., Liverani L., Brucato V., La Carrubba V., Boccaccini A.R. (2018). In vitro degradation and bioactivity of composite poly-l-lactic (PLLA)/bioactive glass (BG) scaffolds: Comparison of 45S5 and 1393BG compositions. J. Mater. Sci..

[45] Fabbri P., Cannillo V., Sola A., Dorigato A., Chiellini F. (2010). Highly porous polycaprolactone-45S5 Bioglass^®^ scaffolds for bone tissue engineering. Compos. Sci. Technol..

[46] Torres F., Nazhat S., Sheikhmdfadzullah S., Maquet V., Boccaccini A. (2007). Mechanical properties and bioactivity of porous PLGA/TiO_2_ nanoparticle-filled composites for tissue engineering scaffolds. Compos. Sci. Technol..

[47] Fu Q., Saiz E., Rahaman M.N., Tomsia A.P. (2011). Bioactive glass scaffolds for bone tissue engineering: State of the art and future perspectives. Mater. Sci. Eng. C Mater. Biol. Appl..

[48] Charles-Harris M., del Valle S., Hentges E., Bleuet P., Lacroix D., Planell J.A. (2007). Mechanical and structural characterisation of completely degradable polylactic acid/calcium phosphate glass scaffolds. Biomaterials.

[49] Leal A.I., Caridade S.G., Ma J., Yu N., Gomes M.E., Reis R.L., Jansen J.A., Walboomers X.F., Mano J.F. (2013). Asymmetric PDLLA membranes containing Bioglass^®^ for guided tissue regeneration: Characterization and In Vitro biological behavior. Dent. Mater..

[50] Avashia Y.J., Sastry A., Fan K.L., Mir H.S., Thaller S.R. (2012). Materials used for reconstruction after orbital floor fracture. J. Craniofac. Surg..

[51] Baino F. (2011). Biomaterials and implants for orbital floor repair. Acta Biomater..

[52] Deng C., Weng J., Lu X., Zhou S.B., Wan J.X., Qu S.X., Feng B., Li X.H. (2008). Preparation and in vitro bioactivity of poly(d,l-lactide) composite containing hydroxyapatite nanocrystals. Mater. Sci. Eng. C.

[53] Zhang R., Ma P.X. (2004). Biomimetic polymer/apatite composite scaffolds for mineralized tissue engineering. Macromol. Biosci..

[54] Lusvardi G., Malavasi G., Menabue L., Menziani M.C. (2002). Synthesis, Characterization, and Molecular Dynamics Simulation of Na_2_O-CaO-SiO_2_-ZnO Glasses. J. Phys. Chem. B.

[55] Courthéoux L., Lao J., Nedelec J., Jallot E. (2008). Controlled Bioactivity in Zn-doped sol-gel derived SiO2 -CaO bioactive glasses. J. Phys. Chem. C.

[56] Linati L., Lusvardi G., Malavasi G., Menabue L., Menziani M.C., Mustarelli P., Segre U. (2005). Qualitative and quantitative structure-property relationships analysis of multicomponent potential bioglasses. J. Phys. Chem. B.

[57] Zhang Z., Dong H., Gorman B.P., Mueller D.W., Reidy R.F. (2004). Behavior of copper ions in silica xerogels. J. Non. Cryst. Solids.

[58] Schreiber H.D., Kochanowski B.K., Schreiber C.W., Morgan A.B., Coolbaugh M.T., Dunlap T.G. (1994). Compositional dependence of redox equilibria in sodium silicate glasses. J. Non. Cryst. Solids.

[59] Hoppe A., Meszaros R., Stähli C., Romeis S., Schmidt J., Peukert W., Marelli B., Nazhat S.N., Wondraczek L., Lao J. (2013). In vitro reactivity of Cu doped 45S5 Bioglass^®^ derived scaffolds for bone tissue engineering. J. Mater. Chem. B.

[60] Láctico L.Á., Motta A.C. (2007). Síntese e Caracterização do Copolímero Poli (L-co-D,L Ácido Láctico). Polimeros.

[61] Coleman N.J., Bellantone M., Nicholson J.W., Mendham A.P. (2007). Textural and structural properties of bioactive glasses in the system CaO–SiO2. Ceram. Silik..

[62] Srivastava A.K., Pyare R. (2012). Characterization of CuO substituted 45S5 Bioactive Glasses and Glass-Ceramics. Int. J. Sci. Technol. Res..

[63] Neščáková Z., Zheng K., Liverani L., Nawaz Q., Galusková D., Kaňková H., Michálek M., Galusek D., Boccaccini A.R. (2019). Multifunctional zinc ion doped sol-gel derived mesoporous bioactive glass nanoparticles for biomedical applications. Bioact. Mater..

[64] Balasubramanian P., Strobel L.A., Kneser U., Boccaccini A.R. (2015). Zinc-containing bioactive glasses for bone regeneration, dental and orthopedic applications. Biomed. Glasses.

[65] Nasrazadani S., Hassani S. (2016). Modern Analytical Techniques in Failure Analysis of Aerospace, Chemical, and Oil and Gas Industries.

[66] Bernstein M., Gotman I., Makarov C., Phadke A., Radin S., Ducheyne P., Gutmanas E.Y. (2010). Low temperature fabrication of β-TCP-PCL nanocomposites for bone implants. Adv. Eng. Mater..

[67] Lei Y., Rai B., Ho K.H., Teoh S.H. (2007). In vitro degradation of novel bioactive polycaprolactone—20% tricalcium phosphate composite scaffolds for bone engineering. Mater. Sci. Eng. C.

[68] Koort J.K., Makinen T.J., Suokas E., Veiranto M., Jalava J., Tormala P., Aro H.T. (2008). Sustained release of ciprofloxacin from an osteoconductive poly(DL)-lactide implant. Acta Orthop..

[69] Bi L., Jung S., Day D., Neidig K., Dusevich V., Eick D., Bonewald L. (2012). Evaluation of bone regeneration, angiogenesis, and hydroxyapatite conversion in critical-sized rat calvarial defects implanted with bioactive glass scaffolds. J. Biomed. Mater. Res. A.

[70] Kikuchi M., Koyama Y., Yamada T., Imamura Y., Okada T., Shirahama N., Akita K., Takakuda K., Tanaka J. (2004). Development of guided bone regeneration membrane composed of beta-tricalcium phosphate and poly (L-lactide-co-glycolide-co-epsilon-caprolactone) composites. Biomaterials.

[71] Albrecht T.W.J., Addai-Mensah J., Fornasiero D. Effect of pH, Concentration and Temperature on Copper and Zinc Hydroxide Formation/Precipitation in Solution. Proceedings of the Chemeca 2011: Engineering a Better World.

